# Attitudes of Infertile Couples, Fertility Clinic Staff and Researchers toward Personhood of The Human Embryo in Iran

**DOI:** 10.22074/cellj.2016.4989

**Published:** 2017-02-22

**Authors:** Marjaneh Kayssan, Mahrokh Dolatian, Reza Omani Samani, Saman Maroufizadeh

**Affiliations:** 1 Department of Midwifery and Reproductive Health, School of Nursing and Midwifery, Shahid Beheshti University of Medical Sciences, Tehran, Iran; 2 Medical Ethics and Law Research Center, Shahid Beheshti University of Medical Sciences, Tehran, Iran; 3 Department of Epidemiology and Reproductive Health, Reproductive Epidemiology Research Center, Royan Institute for Reproductive Biomedicine, ACECR, Tehran, Iran

**Keywords:** Attitude, Personhood, Human Embryo, Ensoulment, Fetus

## Abstract

**Objective:**

After the introduction of assisted reproductive techniques, human embryos were officially introduced into laboratories and now thousands of them are cryopreserved in such settings. Embryonic stem cells and the future application of such cells in the treatment of disease opened the door to further research on human embryos. These developments raise many ethical issues, some of which have religious aspects. The main question is: what is the embryo? Should we consider it a human being? Thus, the purpose of this study was to investigate attitudes towards the personhood of the embryo.

**Materials and Methods:**

In this cross sectional study, 203 infertile patients (n=406), 54 clinic staff and 49 embryo researchers, selected using convenience sampling at the Royan Institute, completed a questionnaire on personhood of human embryo. The questionnaire had been developed following qualitative research and had satisfied face and content validity tests.

**Results:**

At the pre-implantation stage the majority of participants in all three groups considered the human embryo as "not a human being". Also, at the post-implantation stage of development, the majority of infertile couples and clinic staff considered the embryo as "not a human being" but, half the researchers (51%) considered the embryo in this stage as a "potential human". Half of the infertile couples considered the human fetus before ensoulment time (19^th^ week of pregnancy according to the Shiite Islamic scholars) as "not-human being", while more than half of researchers (55.1%) considered it as a "potential human".

**Conclusion:**

Ensoulment time is a major and important border for personhood. Most infertile couples and clinic staff consider the human embryo as "not a human being" but majority of all study participants considered the human fetus to be a complete human after ensoulment time.

## Introduction

The widespread use of assisted reproductive technology (ART) worldwide has led to the birth of millions of children; for instance 1-3% of all births in the United States of America (USA) and Europe (1). According to a Center for Disease Control and Prevention report for 2011, the number of ART cycles in the USA increased from 115,392 cycles in 2002 to 151,923 cycles in 2011 and the number of babies born using ART increased by 34% (2). According to Nachtigall et al. (3), by 2005 about 400,000 blastocysts had been stored in the freezers of clinics in United States. In Japan, about 61,000 frozen embryos are in storage, among which, 15% have no decision about their fate (4).

The occurrence of surplus human embryos is an unavoidable consequence of routine *in vitro* fertilization (IVF). For safety reasons only a limited number of embryos can be transferred into an infertile woman’s womb during a cycle. In almost all fertility clinics the remaining embryos are cryopreserved for later use (5). As a consequence, hundreds of thousands of embryos have been accumulated, generating difficult challenges for patients, physicians, and embryologists. One important consideration is what to do with the extra embryos; donate them for scientific research or training, donate them to other infertile couples for reproductive use, or discard them (6). Recent advances in the field of embryology and developmental biology have opened up a wide variety of potential benefits for human society, (7) with research on embryonic stem cells indicating a bright future for the treatment of incurable diseases (8). As a consequence, the medical community has become very interested in the potential applications of human embryonic stem cells (hESCs) in regenerative medicine (9). However, the application of hESC raises a series of ethical issues, in particular in relation to traditional moral values, and as a consequence hESC research has aroused huge controversy (10). The extent of these controversies is partly dependent on the source of embryonic stem cells (9). Due to the current level of technology, the derivation of hESC for research or medical purpose will inevitably cause damage to embryos (10). Therefore, today research on hESC has involved in addition to biologists, other groups such as: medical science professors, ethicists and politicians (11).

Moral status is a term people use to capture the extent to which “something” should be given the protections and the level of respect that society gives to human being (12). When a creature has moral status, harming and destroying it or restricting it’s freedom becomes wrong (13). When we talk about the human embryo, the difference between the complete human being and a potential human being gives rise to debate. A distinction is drawn between the concept of a complete human being and a human embryo in various cultures and religions, even under Islamic jurisprudence (14).

Whether the pre-implantation embryo has the same moral status as a complete human being is highly correlated to people’s perception of this issue as influenced by their different social, religious and cultural backgrounds. Perceptions of the meaning of life and death similarly varies between different cultures in the world, and these cultural variations profoundly affect the development of bioethics (10). For example, a study in Japan showed the influence of Japanese moral/cultural values and beliefs to be central to the decision-making process and reflected in how the embryos were conceptualized (4).

Attitudes towards the personhood of human embryos differ between different groups of people. For instance, some people believe that an embryo has maximum moral status, which means that it is always morally unacceptable to destroy an embryo. By contrast, other people believe that an embryo has no moral status, which would mean that there are no ethical problems in destroying an embryo and it can be used it for various purposes such as: donation to other patients or for science. Still others believe that the moral status of an embryo falls somewhere in between. These people believe that under some circumstances it might be morally acceptable to destroy an embryo (12, 15). The aim of this study was to investigate the attitudes towards the personhood of human embryos in a sample of infertile Iranian couples, clinic staff and embryo researchers. Results from the study may help policy makers improve rules, regulations and guidelines for embryo donation and embryonic stem cell research.

## Materials and Methods

### Ethical approval

This cross sectional study was approved by the Ethics Committees of Shahid Beheshti University of Medical Science and the Royan Institute, Tehran, Iran. Cases were assured that no personal data would be published and participation was completely voluntarily. Patients were also reassured that participation or non-participation in the study bore no relation to their treatment protocols. Voluntarily completion of the full questionnaire was considered to constitute informed consent.

### Participants

i. Infertile couples (IC): Infertile couples were recruited from patients referred to the Royan Institute, a referral clinic in Tehran for undergoing assisted reproductive techniques, between December 2015 and May 2016. Candidates for third party reproduction were excluded. Consent to participate and ability to read and write in Persian were our only inclusion criteria. Of the 223 eligible couples (446 individuals) who participated in the study, 20 couples (40 individuals) were excluded from the analyses because their questionnaires were incomplete.

ii. Clinic staff (CS): Infertility clinic staff recruited included physicians, nurses and midwives who were directly in contact with patients. Inclusion criteria were free consent to participate in the study, at least 1 years’ experience in the job, and ability to read and write in Persian. Of the 55 eligible clinic staff who participated in the study, only one was excluded from the analyses due to an incomplete questionnaire.

iii. Researchers (RS): Only Iranian researchers with at least 1 years’ experience of working with embryos in the Royan Institute’s research were recruited into the study. Consent to participate was the only inclusion criteria. Of the 55 eligible researchers recruited, 6 were excluded from the analyses because their questionnaires were incomplete.

### Study design

The study was designed as a cross-sectional, descriptive questionnaire survey. Participants were selected using convenience sampling in all three groups. All eligible infertile patients, clinic staff and researchers completed a questionnaire which came with a brief information sheet about the study and the purpose of the investigation. Among the infertile couples, questionnaires were completed separately by women and men.

### Development of questionnaire

Design of the questionnaire involved the following steps:

i. Qualitative research on all aspects of the human embryo was carried out using in-depth interviews with gynecologists, embryologists, law experts, clerics, sociologists, coroners and ethicists. Items were extracted from the interviews using content analysis.

ii. A review of the literature was carried out on attitudes toward human embryos and their moral status. Items were added to those extracted in step 1

iii. A questionnaire was developed on attitudes toward different aspects of the human embryo from the items derived from steps 1 and 2.

iv. Content validity was assessed using 18 experts and academic staff members from Tehran and Shahid Beheshti Universities, the Medical Ethics and History of Medicine Research Center at Tehran University and the Medical Ethics and Law Research Center from Shahid Beheshti University (3 gynecologists, 10 ethicists, 3 coroners, 2 clerics).

v. Face validity was assessed using 20 infertile couples who were asked about the clarity of the questionnaire items and checked by two Persian literature experts for fluency of the text. A graphics expert designed the fonts and appearance of the questionnaire.

### Measures

The questionnaire consisted of two parts. Part one consisted of questions on socio-demographic characteristics and infertility history and part two about attitudes towards personhood of the human embryo during different stages of development from pre-implantation till after ensoulment. Ensoulment time is defined as when the soul enters the fetus that differs between religions. Shiite scholars believe that it happens in 120th day of gestational age. For better understanding by patients, the questions in this part had 5 choices including: a cell mass, human cell mass, living creature, potential human and complete human. On subsequent statistical processing, the 5 options were merged to 3 options in which three choices (cell mass, human cell mass, living creature) were considered as a single category: "not a human being".

### Statistical analysis

In this study, descriptive statistics for continuous variables were presented as mean ± SD and for categorical variables as numbers (percentage). Chi-square test was used to compare groups. Data analysis was carried out using SPSS software version 16.0 (SPSS Inc., Chicago, IL, USA). All statistical tests were 2-sided and the level of statistical significance was set at 0.

## Results

Infertile couples: Demographic characteristics (Table 1): Mean age was 33.27 ± 5.1 among men and 29.25 ± 4.8 among women. The distribution by educational status was 15.4% primary, 37.8% secondary and 46.5% university education among men, and 13.4% primary, 34.3% secondary and 52.2% university education among women. Mean duration of marriage was 6.59 ± 3.6 and duration of infertility was 4.34 ± 3.1. Main religion was Islam 99% which included 3.9% Sunni and 95.1% Shiite. Cause of infertility were 33.4% male factor, 13.1% female factor, 11.8% both and 41.7% unexplained infertility. 20.2% had a history of abortion and 19.7% had a past treatment history. 25.1% of the cases had secondary infertility and only 9.4% of the cases had a previous alive-born child. 19.7% of the cases had a history of treatment failure, of which 10.9% had more than one failure. Only 7.4% had frozen embryos in a fertility clinic.

Clinical staff: Demographic characteristics (Table 2): The mean age of the clinic staff was 35.13 ± 6.60; 74.1% had a bachelor´s degree, 14.8% a master’s degree and 11.1% a Ph.D. Their religion was Islam and 87% were women.

Researchers: Demographic characteristics (Table 2): The mean age of the researchers was 33.92 ± 4.6; 10.2% had a bachelor´s degree, 53.1% a master’s degree and 36.7% a Ph.D. Their main religion was Islam and 63.3% of them were women.

i. Pre-implantation embryos (Table 3): Among infertile couples 83.7% considered pre-implantation embryos as not a human, 11.3% as a potential human and 4.9% as a complete human. Among clinic staff 77.8% considered embryos as not a human, 18.5% as a potential human and 3.7% as a complete human. Similarly among researchers: 67.3% considered embryos as not a human, 30.6% as a potential human and 2% as a complete human. We found significant difference between infertile couples and researchers in all three categories (P=0.001). At this stage of development, although the majority in all three groups considered the embryo as not a human, the proportion of infertile couples with this view was higher than in the other groups, especially the researchers. In other words, researchers were more likely to think of the pre implantation embryo as a potential human than were the infertile couples, and infertile couples were more likely to think of pre-implantation embryos as not a human or a complete human than the researchers.

ii. Post-implantation embryos (Table 3): Among infertile couples 72.3% considered post-implantation embryos as not a human, 19% as a potential human and 8.6% as a complete human. Among clinic staff 66.7% considered embryos at this stage as not a human, 27.8% as a potential human and 5.6% as a complete human. Similarly among researchers: 44.9% considered pre-implantation embryos as not a human, 51% as a potential human and 4.1% as a complete human. We found significant difference between infertile couples and researchers in all three categories (P<0.001). In the second stage of development, the majority of infertile couples and clinic staff considered the embryo as not a human; however, the proportion of infertile couples reporting this view was higher than in the other groups. At this stage of development, half of the researchers (51%) considered the embryo as a potential human, a percentage which was higher than that among infertile couples. Conversely the proportion of researchers who considered the post-implantation embryo to be not a human or a complete human was lower than infertile couples.

iii. Fetus prior to ensoulment (Table 3): Among infertile couples 50% considered the fetus prior to ensoulment as not a human, 27.6% as a potential human and 22.4% as a complete human. Among clinic staff 46.3% considered the fetus prior to ensoulment as not a human, 42.6% as a potential human and 11.1% as a complete human. Among researchers 28.6% considered the fetus prior to ensoulment as not a human, 55.1% as a potential human and 16.3% as a complete human. We found significant difference between infertile couples and researchers in all catgories (P<0.001). At this stage of development in which the fetus is complete from a bodily point of view, but hasn’t been ensouled, half of the infertile couples conceived fetus as not a human, compared with more than half of the researchers (55.1%) who considered it as a potential human.

iv. After ensoulment (19th week) (Table 3): Among infertile couples 17% considered the fetus post-ensoulment as not a human, 24.4% as a potential human and 58.6% as a complete human. Among clinic staff 13% considered the fetus post-ensoulment as not a human, 20.4% as a potential human and 66.7% as a complete human. Among the researchers: 12.2% considered the fetus post-ensoulment as not a human, 16.3% as a potential human and 71.4% as a complete human. At this stage of development, we did not found any significant difference between the categories for any of the three groups of participants (P=0.413). In all three groups the majority of participants agreed that the fetus post-ensoulment is a complete human and only a few of them thought of the fetus post-ensoulment as not a human.

**Table 1:** Socio-demographic characteristics of the infertile couples


	FemaleMean ± SD or n (%)	MaleMean ± SD or n (%)

Age (Y)	29.25 ± 4.83	33.27 ± 5.14
Duration of marriage (Y)	6.59 ± 3.68	-
Duration of infertility (Y)	4.34 ± 3.178	-
Education		
Primary	27 (13.4)	31 (15.4)
Secondary	69 (34.3)	76 (37.8)
University	105 (52.2)	94 (46.5)
Ethnicity		
Fars	123 (61.2)	114 (56.7)
Turk	44 (12.9)	45 (22.4)
Kurd	23 (11.4)	29 (14.4)
Lor	9 (4.5)	11 (5.5)
Others	2 (1.0)	2 (1.0)
Religion		
Islam- Shiite	193 (95.1)	192 (94.6)
Islam- Sunni	8 (3.9)	9 (4.4)
Christian	2 (1.0)	2 (1.0)
Cause of infertility		
Male factor	133 (33.4)	-
Female factor	52 (13.1)	-
Both	47 (11.8)	-
Unexplained	166 (41.7)	-
History of abortion		
Yes	41 (20.2)	-
No	162 (79.8)	-
Type of infertility		
Primary	152 (74.9)	-
Secondary	51 (25.1)	-
Number of children from natural pregnancy		
Zero	184 (90.6)	-
One	19 (9.4)	-
IVF/ICSI treatment history		
Yes	40 (19.7)	-
No	163 (80.3)	-
The number of unsuccessful treatments		
Zero	163 (80.3)	-
One	18 (8.9)	-
Two and more	22 (10.9)	
Have cryopreserved embryos		
Yes	15 (7.4)	-
No	188 (92.6)	-
Number of embryos		
Zero	188 (92.6)	-
One or more	15 (7.4)	-
Number of children from IVF/ICSI		
Zero	197 (97)	-
One	6 (3.0)	-


IVF; In vitro fertilization and ICSI; Intr-cytoplasmic sperm injection.

**Table 2:** Demographic characteristics of the clinic staff and researchers from the Royan Institute


	Clinic staff (n=54) Mean ± SD or n (%)	Researchers (n=49) Mean ± SD or n (%)

Age (Y)	35.13 ± 6.68	33.92 ± 4.69
Gender		
Male	7 (13.0)	18 (36.7)
Female	47 (87.0)	31 (63.3)
Education		
Bachelor’s	40 (74.1)	5 (10.2)
Master’s	8 (14.8)	26 (53.1)
Ph.D.	6 (11.1)	18 (36.7)
Ethnicity		
Fars	51 (94.4)	39 (79.6)
Turk	2 (3.7)	8 (16.3)
Others	1 (1.9)	2 (4.1)
Religion		
Islam- Shiite	54 (100.0)	48 (98.0)
Zoroastrian	0 (0)	1 (2.0)
Work and research experiences (Y)	8.48 ± 6.60	6.90 ± 3.99
Field of study		
Physician	5 (7.8)	0 (0)
Midwife	30 (56.3)	0 (0)
Nurse	19 (35.9)	0 (0)
Cell and molecular biology	0 (0)	49 (100.0)


**Table 3:** Attitude of infertile couples, fertility clinic staff and researchers toward personhood of human embryo


	Infertile couple n=406	Clinic staffn=54	Researchern=49	P value	Pairwise significance differences

Personhood of pre-implantation embryos				0.005	IC and RS
Not human	340 (83.7)	42 (77.8)	33 (67.3)		
Potential human	46 (11.3)	10 (18.5)	15 (30.6)		
Complete human	20 (4.9)	2 (3.7)	1 (2.0)		
Personhood of post-implantation embryos				<0.001	IC and RS
Not human	293 (72.3)	36 (66.7)	22 (44.9)		
Potential human	77 (19.0)	15 (27.8)	25 (51.0)		
Complete human	35 (8.6)	3 (5.6)	2 (4.1)		
Personhood of fetus				<0.001	IC and RS
Not human	203 (50.0)	25 (46.3)	14 (28.6)		
Potential human	112 (27.6)	23 (42.6)	27 (55.1)		
Complete human	91 (22.4)	6 (11.1)	8 (16.3)		
Personhood of fetus after ensoulment				0.413	-
Not human	69 (17.0)	7 (13.0)	6 (12.2)		
Potential human	99 (24.4)	11 (20.4)	8 (16.3)		
Complete human	238 (58.6)	36 (66.7)	35 (71.4)		


IC; Infertile couples, CS; Clinical staff, and RS; Researchers.

## Discussion

This study examined the attitudes of participants toward the personhood of the embryo, because different social, religious and cultural factors have high impact on people’s perception of the philosophy of life and thus to the moral status of human embryo (10). People' awareness about the moral status of the embryo is considered to be the most powerful predictor of their decision regarding the fate of surplus embryos (16). Taking the first three stages of development together, our findings show that the attitude of participants gradually changes from categorizing these stages as not a human to a potential human, and eventually a complete human but, there were statistically significant differences between the attitudes of the infertile couples and researchers at these three stages. On the other hand, although the tendency to considering the embryo or fetus as a complete human gradually increased by stage of development, it had the least acceptance compared with the other options. At these three stages, the attitude of clinical staff was intermediate between two other groups, but the differences were not statistically significant.

Our findings showed that considering the embryo or fetus as not a human was higher than considering it a potential human during these three stages among infertile couples and clinical staff, but researchers only considered the pre implantation embryo as not a human and after that stage they considered it a potential human. Although there were differences in attitude between the groups toward the personhood of human embryo, these differences only lasted until fourth stage, the fetus post-ensoulment. Most of participants considered the fetus after ensoulment as a complete human. Religious beliefs are the strongest reason for agreement at the fourth stage, ensoulment time, that the fetus is a complete human. Considering the descriptive statistics presented in tables 1 and 2, more than 99% of infertile couples and 98-100% of the other groups were Muslims and according to Islamic authorities there is significant difference between before and after ensoulment. Most of Islamic scholars believe that ensoulment happens after the fourth month of pregnancy (120 days after fertilization). In fact, the human personality begins from that time, in other words, the embryo and fetus become complete human (7). In our findings, we see that consideration of the fetus as a complete human reaches its maximum at the end of the fourth month, and may explain the relationship between the moral status of the embryo and the importance of religious beliefs, as has been shown in other studies (16, 17).

Our data show that only 4.9% of the infertile patients, 3.7% of the clinic staff and 2% of the researchers considered the pre-implantation embryo as a complete human. Blaževičienė et al. (18) reported 68.5% of fertile women and 35.5% of infertile patients perceived the embryo as a human being. Also, Provoost et al. (17) and Lyerly et al. (6) found 30.9 and 18% of infertile couples had given the pre-implantation embryo the moral status of a child or person. Mohler-Kue et al. (16) showed 50.4% of patients agreed that an embryo should be afforded the same dignity and rights as a human being and 27.7% of Americans who participated in the study of Hudson et al believed that an embryo in a petri dish has maximum moral status (12). In most studies, patients believed that the embryo should be valued and respected as a complete human, but in our study only a few participants agreed with this view, making our findings inconsistent with those of previous studies in which the participants were mainly Christians. Our results showed that 11.3% of infertile couples, 18.5% of clinic staff and 30.6% of researchers considered the pre-implantation embryo as a potential human. This is in contrast to the study of Jin et al, in which 33.3% of patients with children 0-3 years old, 48.6% with children 3-5 years old, and 50% with children 5 years old considered the embryo to be a potential child (19). McMahon et al. (20) showed that 51% of cases thought of their embryo as potential children. Our finding also showed that 83.7% of infertile couples, 77.8% of clinic staff and 67.3% of researchers thought of the pre-implantation embryo as not a human. This is contrast to the studies of Wånggren et al. (29.5%) (12), Mohler-Kue et al. (12.1%) (16), Lyerly et al. (10%) (6) and Provoost et al. (43.9%) (17). As can be seen there are obvious differences between our findings and other studies.

In most studies, the views assessed have been those related to moral status and respect for the embryo. For instance, in the study of Mohler-Kue et al. (16), patients’ views on the status of the early embryo were assessed using a 4-point scale which ranged from some respect to the same status as a complete human being. While few participants believed that the embryo is nothing but cluster of cells, only 50% felt the embryo has the same rights as a human. In the study of Lyerly et al. (6), participants’ attitudes were assessed using a 7-point scale in which 1 meant no moral status and 7 meant maximum moral status. The majority of the participants in Lyerly et al.’s study chose intermediate scores. In our study we examined the personhood of the embryo using a 5-point scale that ranged from ‘a mass cells’ to ‘a complete human’.

In some studies, such as that of Jin et al. (19) and Blaževičienė et al. (18), only the attitude of "infertile people" towards embryos as potential humans has been examined and no other choices (like "human cells" or "complete human being") were presented to the patients. Another difference between this study and the others, is that according to study of Jin et al. (19), participants’ attitude towards their cryopreserved embryos is changed by having a child through the IVF treatment and bringing them up, but in this study, most of the couples didn’t have any children. Other studies have examined attitudes of patients to their own cryopreserved embryos and should decide their fate. In other words, participants in those studies were potential embryo donors, while in our survey, the majority of couples did not have any cryopreserved embryos and most of them were at the beginning of the treatment so, maybe, at the time of study, they did not had thought deeply about the personhood of the embryo. This may be one of the reasons that our findings are different from those of other studies. Evidence suggests that people’s intentions regarding excess embryos change after IVF treatment (21).

## Conclusion

There are significant differences between the attitudes of infertile couples, fertility clinic staff and researchers toward the personhood of human embryos in the various stages of development, which are maintained up to ensoulment time (19^th^ week). After the nineteenth week of pregnancy, the majority of participants in all three groups considered the fetus as a complete human.

Most infertile couples and clinic staff consider the embryo as "not a human" until the ensoulment, but more than half of the researchers consider it as a "potential human" except before implantation. Our results show that using excess embryos for treatment and research is likely to be less controversial among Iranian patients and fertility clinic staff than among the researchers themselves. However more extensive studies are need to confirm these findings.
